# Genetically modified organisms and food security in Southern Africa: conundrum and discourse

**DOI:** 10.1080/21645698.2020.1794489

**Published:** 2020-07-20

**Authors:** Norman Muzhinji, Victor Ntuli

**Affiliations:** a Department of Natural and Applied Sciences, Namibia University of Science and Technology, Windhoek, Namibia; b Department of Biology, National University of Lesotho, Roma, Lesotho

**Keywords:** Genetic engineering, GMOs, food security, Southern Africa

## Abstract

The importance of food security and nourishment is recognized in Southern African region and in many communities, globally. However, the attainment of food security in Southern African countries is affected by many factors, including adverse environmental conditions, pests and diseases. Scientists have been insistently looking for innovative strategies to optimize crop production and combat challenges militating against attainment of food security. In agriculture, strategies of increasing crop production include but not limited to improved crop varieties, farming practices, extension services, irrigation services, mechanization, information technology, use of fertilizers and agrochemicals. Equally important is genetic modification (GM) technology, which brings new prospects in addressing food security problems. Nonetheless, since the introduction of genetically modified crops (GMOs) three decades ago, it has been a topic of public discourse across the globe, conspicuously so in Southern African region. This is regardless of the evidence that planting GMOs positively influenced farmer’s incomes, economic access to food and increased tolerance of crops to various biotic and abiotic stresses. This paper looks at the issues surrounding GMOs adoption in Southern Africa and lack thereof, the discourse, and its potential in contributing to the attainment of food security for the present as well as future generations.

## Introduction

One of the biggest challenges that faces humanity in the 21st Century is food insecurity. According to,^1^ more than 800 million people globally lack adequate food and at least 10% of global food production from crops is lost due to unfavorable weather conditions, pests and diseases.^2^ Amid the aforementioned factors, population growth in sub-Saharan Africa is pushing crop production into marginal areas with little and unreliable rainfall, with only 4% of cropland irrigated.^3^ Collectively, this is affecting the wellbeing of many communities including the Southern African Development Community (SADC) countries, which are reliant on agricultural production for their livelihoods.^4^

The SADC is a regional trade grouping comprising 16 Southern African countries: Angola, Botswana, Comoros, DRC, Eswatini, Lesotho, Madagascar, Malawi, Mauritius, Mozambique, Namibia, Seychelles, South Africa, Tanzania, Zambia and Zimbabwe. SADC regional bloc is characterized by rising population growth, diminishing arable land, increased malnutrition and frequent occurrence of natural disasters such as droughts and floods that have left the region food insecure.^5^ Faced with such challenges, strategies have to be formulated, adopted and implemented to ensure the poor and vulnerable communities in SADC, have access to resilient technologies that result in sustainable increase in production of nutritious food. Innovations in crop genetic improvement technologies like genetic engineering have the potential to offer increased, robust sustainable agricultural production in the face of population growth, climate change and shrinking natural resources.^6^ However, many promises of the technology that could have an impact on food security in SADC countries have not been realized because very few countries in the region have fully operationalized the necessary biosafety framework to regulate products of modern biotechnology. Most countries in SADC region have rather adopted a precautionary approach toward regulating GMO foods and crops irrespective of food shortages due to low agricultural production.

## Food Security in Southern Africa

Food security implies the physical, social and economic access to sufficient, safe, nutritious food to maintain a healthy and active life by all people.^7^ In light of this definition, food insecurity, under-nutrition and malnutrition are currently among the most serious concerns affecting many people in SADC region and other developing nations.^8^ Based on the results of SADC vulnerability assessment and analysis report, approximately 41 million people are food insecure and nine million are in urgent need of food aid.^9^ There is, therefore, an urgent need to increase production of nutritious food in SADC countries to avert hunger and malnutrition. Pursuant to that goal, the African Union, to which SADC countries belong, launched the Maputo Declaration on agriculture and food security in 2003 that stipulated that 10% of national budgetary resources of member countries should be committed to increase agricultural production.^10^ Furthermore, in an attempt to improve access to food in the region, the SADC countries through its Dar es Salaam Declaration on Agriculture and Food Security encouraged member countries to prioritize the development of improved crop varieties as a means of improving food security and poverty alleviation in the region.^11^ In 2014, AU member countries reiterated the call of ensuring food security by launching the Malabo Declaration on accelerated agricultural growth and transformation for shared prosperity and improved livelihoods.^12^ The declaration underscored the need to utilize a broad portfolio of tools and technology interventions, including modern biotechnology, to eradicate hunger and malnutrition and to achieve robust sustainable agriculture.

## Challenges and Opportunities for Addressing Food Security in Southern Africa

Attainment of food security in Southern Africa is affected by several factors which include population growth, pest and diseases, over-reliant on rain-fed farming, droughts, and climate change.^2^ These factors are severely militating against the attainment of the United Nations’ Sustainable Development Goals (SDG) of eradicating poverty and hunger (SDGs 1–3).

### Population Growth

The SADC region is characterized by high population growth rate, and as of 2018, the population of Southern Africa was estimated to be 345 million.^5^ Population growth results in increased pressure on resources, leading to high demand for food. Consequently, this presents a massive challenge on agriculture to feed the growing population with nutritious and sufficient food in a sustainable way. Even though SADC supports efforts from member countries to ensure sustainable access to safe and adequate food at all times, the region is failing to cope with the increasing population growth using conventional strategies in agriculture production. Owing to this, there is a continual importation of food from other regions to suffice this challenge.^9^ There is therefore, an urgent need to formulate and adopt strategies and policies that will reconcile the growing food demands of an ever-increasing population in the face of climate change, drought, pest and diseases.

### Pests and Diseases

In SADC region, farmers are losing crops due to the devastating effect of plant pathogens such as insects, bacteria, fungi, and viruses; either in the field or post-harvest. In 2017, all countries in SADC except Lesotho and Mauritius were ravaged by fall armyworm *Spodoptera frugiperda* (J.E. Smith),^13^ (Lepidoptera: Noctuidae)^14^ and tomato leaf miner (*Tuta absoluta*). The pests are reported to cause potential yield losses of between 15%–73%^14^ and 100%, respectively.^15^ In addition, the effect of emerging pests and diseases like the maize lethal necrosis disease and cassava brown streak virus has been heavily felt on crops in Tanzania and Zambia, respectively.^16,17^ Crop losses due to pests and diseases are increasingly becoming more common, mostly driven by extreme weather events, pathogen drift, and transboundary movement of plant material.^2^ The control of crop pathogens relies on the use of herbicides and pesticides, but these are not environmentally friendly and economical to smallholder farmers who constitute the majority of producers in Southern Africa. Furthermore, the use of chemicals creates pesticide resistance, which can be detrimental to sustainable management of diseases. Studies have documented that genetic engineering technology together with other strategies can be used to address problems of crop diseases.^18^ For example, the Bt maize, genetically engineered with *Bacillus thuringiensis* (Bt) gene, provides resistance to important insect pest especially the European corn borer and other lepidopteran maize pests.^18,19^ In South Africa, more than 80% of maize planted is Bt maize^20^ and it has been reported to significantly reduce pesticide usage and crop damage.^21^ Furthermore, Bt maize is less susceptible to fungal attack and this ultimately improves food safety by greatly reducing mycotoxin levels in field crops.^22^

### Climate Change Variability

In addition to population growth, pests, and diseases, agricultural production in SADC region has been affected by recurrent droughts and extreme weather conditions like flooding and tropical cyclones. In 2016, 29 million people were reported to be food insecure as a result of the El Nino induced drought that had serious devastating consequences in the region and left many people in need of food aid.^23^ In 2018, an estimated 3.8 million people were affected in Comoros, Malawi, Mozambique and Zimbabwe due to cyclone Idai.^24^ Due to climate change variability, crop growing seasons are now characterized by delayed onset and erratic rainfall, mid-season hot dry spells and early cessation of the rainy season leaving most smallholder farmers vulnerable to food shortages. Forecasting ahead, climate change variability is projected to increase and become more severe over the next decades.^4^ Genetic engineering technology can produce drought, salt, waterlogging-tolerant crops with the potential to be cultivated in areas that lack quality cultivatable land.^25^ This could stabilize and increase food supply, which is important against the background of inexorable rise in food demand, climate change, land and water scarcity.^26^ Confined field trials of the TELA Maize Project, a progression of the Water Efficient Maize for Africa (WEMA), have shown promise of developing drought tolerant and insect resistant hybrid maize for small holder farmers of sub-Saharan Africa.^27^

### Malnutrition

In Southern Africa, malnutrition is a major challenge especially to children and women with a proportion malnutrition population reaching up to 45%. The potential of GM technology as a tool to increase nutritionally enhanced foods cannot be overemphasized. Biofortified GM crops with enhanced vitamin content and better nutritional protein qualities like the Golden Rice have been developed and approved in countries like Philippines increasing micronutrient availability.^28^ Similarly, genetic engineering technology can provide cost-effective food fortification with minerals and vitamins that can solve problems of malnutrition in Southern Africa.^29^ This is especially vital in Southern Africa where food is not abundant, and the choices are not varied.

### Farming Practices and Cost of Production

The majority of the population (60%) in the SADC region are small-scale farmers leaving in rural communities where they depend on subsistence farming for their livelihood.^3,9^ Agricultural production in such communities relies on seasonal rainfall and traditional farming methods that expose them to the vagaries of climate change. In addition, rural farmers are poorly resourced and cannot afford expensive fertilizers and agrochemicals. Eventually the smallholder farmers expend a lot of energy in futile farming when they can take advantage of modern technologies. In countries like South Africa, China, India, Colombia, Brazil, cultivation of GMOs has helped farmers grow more food and feed using fewer resources and reduced cost of pest and weed control.^30–32^ Several studies have shown that GMOs adoption reduces chemical pesticide use and increases yields in farmers’ fields which translate into the cost savings in terms of labor and insecticides as well as environmental benefits.^25^ In China and India, farmers using Bt cotton reduced pesticide use by 8.4%, resulting in substantial economic benefits for smallholder farmers.^33^ A desktop analysis of GMO published articles from 1995 to 2014 conducted by,^34^ found that the adoption of GM technology has reduced the use of chemical pesticides by 37%, and increased farmer profits by 68%. There are also documented health benefits for farm workers because of less chemical pesticide spraying.^35^ Dramatic reductions in pesticide poisonings were reported among Chinese cotton farmers and among cotton farmers in India.^36,37^

## Scientific Innovation Progress and Food Security

Given the issues and challenges facing agricultural systems in Southern Africa, an integrated approach is required to position agricultural systems toward sustainability, food security and attainment of SDGs. These approaches include but are not limited to breeding of high yielding crop varieties with desired characteristics, conservation agriculture, modern biotechnology and use of agrochemicals and fertilizers.^33,38^ Thus far, no approach has proven to be a quick and easy solution to the challenges of food insecurity and sustainable agriculture in Southern Africa.^39^ However, holistic adoption of different approaches will transform agricultural systems in Southern Africa and ensure greater food security as has been demonstrated in other countries like the USA^34^ and China,^40^ where different forms of sustainable agriculture have coexisted successfully.^41^ In these countries, scientific innovations including biotechnology have delivered substantial agronomic, environmental, and economic, health and social benefits to farmers, and to the consumers.^41,42^

### Pre-Mendelian Breeders

In the distant past, farmers (pre-Mendelian breeders) ensured food security by intuitive selection of crops with desirable characteristics (disease resistance, size, yield, and color) and used them as seed source for subsequent generations.^43,44^ However, this did not keep pace with population growth, depletion of soil nutrients, plant pathogens and pests, unreliable rain-fed farming and high post-harvest losses, which left many households food insecure.^43^

### Post-Mendelian Breeding and Controlled Mating

Challenges of crop productivity led to transformation of farming methods with farmers, breeders and scientists relentless finding ways of developing new crop cultivars that are higher yielding, more nutritious, disease-resistant and climate-smart.^44^ Conventional breeding is based on the laws of inheritance and promoting recombination of favorable alleles to generate many plant genotypes. Conventional plant breeding has resulted in high yielding varieties, but challenges of phenotype selection, inbreed depression, heterozygosity and inherently long generation times in some crops have hampered breeding programs of many crops.^26^ Advances in genetic technologies and bioinformatics tools have opened new opportunities, which scientists and plant breeders can utilize to produce high yielding crop varieties in a relatively short period of time.^40^ These technologies include genotype-by-sequencing, marker-assisted selection, and speed breeding.^45,46^

Some methods utilizing chemicals or radiation to introduce mutational variation in crops, which have beneficial traits to farmers have been used. However, these depend on random chance and so often introduce unintended effects. Modern biotechnology is considered a very important tool for circumventing some of the barriers ingrained in conventional breeding methods.^47^

## GM Technology in Southern Africa: The Opportunity?

Agricultural biotechnologies offer an unprecedented capability to greatly accelerate improvements in crops, especially for traits that are lacking in crop genomes. Genetic modification of crops can improve nutritional quality and reduce the need for agricultural inputs such as fertilizers, pesticides, and water, which is particularly useful for smallholder farmers who may not have easy access to these inputs.^48^ GMOs are living organisms whose genetic material has been artificially altered in a laboratory through genetic engineering to give it a characteristic that it does not possess naturally.^46^ Genetic engineering allows for alteration of just a few genes within a plant that has tens of thousands of genes making it faster and precise than conventional breeding.^26^ The growth of modern biotechnology in Southern Africa’s agricultural systems has been limited by uncertainties pertaining to the perceived effects of GMOs on health and environment.^29,49^ This has unfortunately downplayed potentially valuable opportunity for crop improvement for pesticide resistance, drought tolerance and nutritional improvements, where new developments are most needed to enhance food security.

## Biosafety Laws and GM Cultivation in Southern Africa

**Figure 1.**
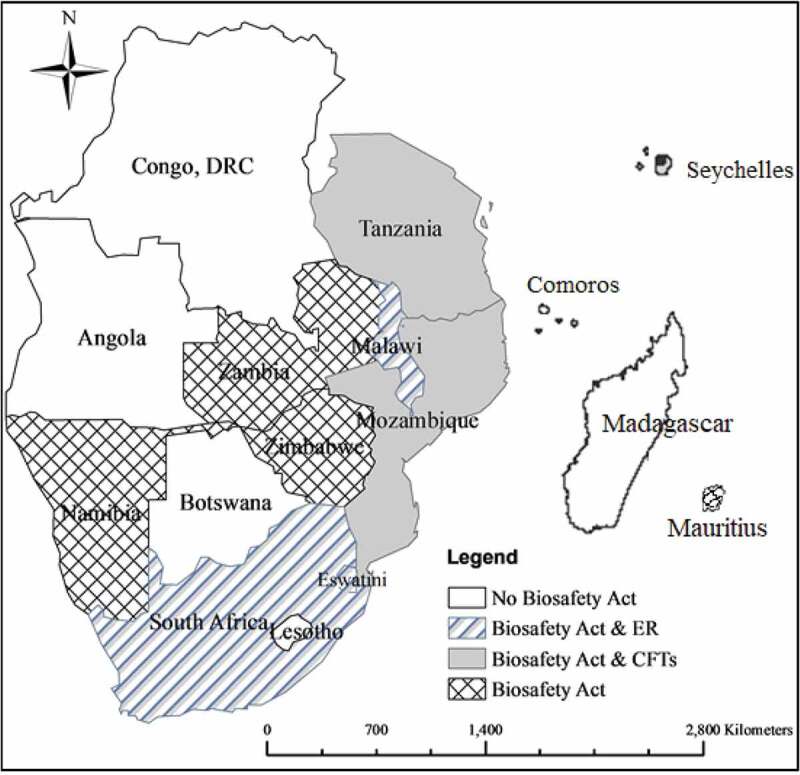
Status of National biotechnology framework and legislation in Southern Africa countries. Source: ABNE ^51^ with modifications.

The Cartagena Protocol on Biosafety (CPB) is a supplementary instrument to the Convention on Biodiversity (CBD). Through its Article 21, it acknowledges the potential of biotechnology in addressing many environmental and developmental problems, including enhancing food security.^50^ Fundamental to the effective implementation of the CBD protocol is the need for Parties to have capacity for proper and safe management of biotechnology as per Article 22 of the Protocol. To that end, all the 16 countries that form the SADC economic bloc are signatories to the CPB. However, countries are at different levels (Figure 1) of domesticating the provisions of the CPB protocol and therefore adoption of GMOs.^52^ South Africa is the only country in SADC region that has taken the permissive principle and has been growing, consuming and trading in GMOs since 1997.^31^ In contrast, other SADC countries have taken the precautionary principle, a risk-oriented approach, that takes into consideration perceived risks and the unknown consequences GMOs might bring to health and the environment.^53^ The precautionary principle forms the basis of the protection of rights of local communities, farmers and breeders and for the regulation of access to biological resources, and the African Model Law on safety of biotechnology.^54^ However, some authors argued that this justification is based upon a selective application of the principle ignoring the enormous benefits associated with GM technology. Contrastingly, South Africa and other countries that support GMOs such as the USA occasionally donate food in the form of drought relief aid.^55^ In addition, South Africa also exports a substantial amount of GM maize and maize-seed to its neighboring countries in the SADC region and abroad.^56^

*Mauritius has a Biosafety act*Comoros, Madagascar and Seychelles does not have a Biosafety act*ER – Environmental commercial release*CFTs – Confined field trials

GMOs adoption in Southern Africa mirrors the progress made by countries in domesticating the CPB protocol (Figure 1). Countries like Lesotho and Zambia prohibit the cultivation and imports of GMOs^41^ whereas Angola and Zimbabwe prohibit cultivation but allow only imports of GMO products.^52^ Malawi and Eswatini recently approved commercial release of GMOs while Mozambique and Tanzania have been conducting confined field trials of GMOs crops (ABNE ^51^ Figure 1; Table 1).

**Table 1.** Status of GMO adoption in Southern African region. Source: ABNE ^51^.

Country	Crop	Trait	Regulatory status
Lesotho	-^a^	-	no CFT and ERs
Comoros	Sugarcane	Batsa tolerance	Research
Mauritius	Sugarcane	-	Research
Seychelles	-	-	-
Malawi	Banana	Viral resistance	CFTs^b^
	Cowpea	Insect resistance	CFTs
	Cotton	Insect resistance	CFTs, ER^c^
Mozambique	Maize	Drought tolerant	CFTs
Tanzania	Maize	Drought resistant	CFTs
	Cassava	Virus resistant	Research
Madagascar	-	-	No research No CFTs No ERs
Eswatini	Cotton	Insect resistance	ER
South Africa	Maize	Insect resistant Herbicide tolerant	ER
		Insect/Drought tolerant	ER
	Cotton	Herbicide tolerant	ER
	Soybean	Herbicide tolerant High allelic acid content	ER
	Oil seed rape	Herbicide tolerant	ER
	Rice – *Oryza sativa*	Herbicide tolerant	ER
	Potato	Insect resistant	CFT
Zambia	-	-	No research, No CFTs No ERs
Democratic Republic of Congo	-	-	No research, No CFTs No ERs
Namibia	-	-	No research, No CFTs No ERs
Botswana	-	-	No research, No CFTs No ERs
Angola	-	-	No research, No CFTs No ERs
Zimbabwe	Cotton	Insect resistant	CFTs (abandoned 2002)

^a^(-) – Nothing to report

^b^CFTs – Confined field trials

^c^ER – Environmental commercial release

In the past few years, GM research, confined field trials and environmental release have been steadily increasing in SADC countries (Table 1). South Africa is one of the countries in the world with accumulated evidence on the benefits of GM crop cultivation.^42^ The benefits include reduced input costs for farming, conservation of the ecosystem and stress-tolerant crops.^25^

## GMO Safety and Politics-The Debate at Crossroads

A total of 70 countries adopted GM crops through cultivation and importation as of 2018, an indication of the importance of GM crops in meeting the global challenges of food insecurity, malnutrition and climate change. Despite the extensive cultivation of GMOs across the globe their safety remains the major topic of GMO discourse.^42^ Most of the reasons against GMOs are scientifically plausible though they are more speculative in nature and not supported by empirical and objective evidence. Taking this into perspective, unlike crops derived from selective breeding, inter-species crossing, scientists know which genes are affected when they splice a specific gene for a desired trait into a crop.^18^ Modern genetic modification affects only a handful of genes, compared to tens of thousands that are affected by less sophisticated conventional breeding methods.^57^

The GM products have been on the market for over 23 years, and there have not been reported cases of food or feed safety issues ever associated with the technology. There is little documented evidence, albeit controversial^58^ that shows GM crops are potentially unsafe. In their review,^59^ reported that GM crops don’t pose significant hazards to the environment and human health. Before environmental release, GM crops are subjected to rigorous risk-assessment and risk-management measures to evaluate risks to human health (including toxicity and allergenicity), risks of evolution of resistance in target pathogens or pests, risks to non-target organisms and risks from movement of transgenes.^60^ These science-based regulatory reviews and risk assessment conducted by,^61^ in Norway for GMO maize 1507 revealed that the modified maize is nutritionally equivalent to conventional maize varieties. In addition, they found no evidence of adverse effect on the environment and health reaffirming the safety of GMOs and the concept of substantial equivalence.

In addition to fears of GMO safety to humans and the environment, issue of intellectual property rights, trade, biodiversity, NGO lobbying and the CBP protocol have hindered the adoption of GMOs in SADC countries . With respect to property rights, there are concerns that GM crops would replace conventional varieties and therefore make farmers depend on private seed companies like Corteva, Syngenta and Bayer who will have the oligopoly to control food supplies. This will interfere with the roles of indigenous farmers as custodians of agricultural biodiversity. There are also concerns related to the impacts of GMOs on biodiversity especially on gene flow, effects on non-target species. The existing capacities of SADC countries to undertake research and effectively monitor and evaluate the impact of GM products on biodiversity is questionable.^62^ With respect to trade, the global adoption of GMOs has been heterogeneous with much resistance observed in the EU countries the major trading partner of SADC countries.^52,57^ Any attempt to allow GMO products could mean loss of product export sales to the EU market.

## Socio-Economic Considerations of the CPB Protocol

CPBP protocol came into effect in 2003 after protracted negotiations since 1997, and all the 16 SADC countries are signatory to the protocol. The CPB showed some potential as an international agreement for regulating GMO. However, it evolved into an agreement not based strictly on scientific risk assessments, but also allows for consideration of socio-economic issues (SECs) annunciated on Article 26 of the Protocol. Article 26 of the CPB protocol encourages countries to take into consideration the socio-economic impact of adoption of GMOs. This use of the precautionary principle in addition to ambiguous interpretations of how to evaluate SECs have been a major barrier to adoption of GMOs. The issues to consider under SECs in SADC are naturally subjective, as ethical issues are not universally defined and a by-product of cultural heritage.^63^

## The GMO Trajectory

After 30 years of cultivation, GMO crops are now well established. The global cultivated area of transgenic crops has increased more than a hundred fold, from 1.7 million hectares in 1996 to 191.7 million hectares in 2018.^42^ Furthermore, 30% of the area under GMO is in developing countries, where the recent rate of increase has been higher than in industrialized countries. Studies on the evidence GMO safety are also increasing.^64^ Growers in Southern Africa may have to start changing the way they grow agricultural crops and start transforming their approaches in line with the Malabo Declaration, if agriculture is to survive and become more sustainable. There are no easy solutions to the impacts of climate change. Scientists can, however, contribute in an important way to the more tangible issue of food security.

The most recent report of sweet potato that contains expressible bacterial DNA is likely to change the paradigm on the safety perceptions of GMOs.^65^ Scientists using high through-put next generation sequencing (NGS) technologies found bacterial genes in sweet potato varieties, commonly grown and consumed in USA, Indonesia, China, South America and Africa.^65^ The research found that the bacteria inserted the genes into the crop’s wild ancestor, long before humans started eating sweet potatoes. Therefore, the first GMO was made by nature. Humans have therefore been eating GMO’s for thousands of years unknowingly. The natural GM sweet potato might be helpful for regulators and scientists looking at the safety of GM crops to think differently. The study puts the GMO technology and safety debate into context because ideally nothing is artificial, scientists are just putting their foot to the accelerator of a natural process.

The success of continued commercial production of the GM crops in most countries including South Africa bears testimony to the value of GM technology in partially addressing complex challenges facing food security and sustainability in the world. It is difficult to predict the origin of the next plant disease catastrophe that will affect one of the most important food crops vital to food security in some parts of the globe. However, there is inadequate control of known diseases in many parts of the world today and in the future. There will be some unpleasant shocks from emerging and re-emrging pathogens that have evolved new virulence characteristics, induced by climate change effects evolutionary factors and global spread (akin to COVID-19 pandemic).

## New Opportunities- Genome Editing

The technological revolution in genomics-based agriculture presents opportunities for a more efficient and precise method for genetic manipulation of crops. The Clustered Regularly Interspaced Short Palindromic Repeats (CRISPRs- CAS9), a technique that has been trending fast, is likely to revolutionize aspects of genetic modification.^66,67^ Genome editing using CRISPRs – CAS9, allows much smaller changes made to DNA compared with conventional genetic engineering.^68^ The process is currently being used in a variety of applications to adapt the DNA of crops to improve their growth characteristics for particular climates or to help them be less susceptible to diseases. For example, scientists in China recently reported creating a strain of wheat that is resistant to powdery mildew, a destructive fungal disease.^69,70^ As the world undergoes many changes in the coming years relating to food insecurity, it will be the turn of scientists and researchers to supply solutions for not only to increase crop productivity but also for socio-economic and climate-related issues.

## Conclusion

Failure to adopt GM technology based on SECs or the precautionary principle is not hurting the scientist, the politician nor the policy maker but the poor peasant farmer, who expend a lot of energy toiling in infertile and unproductive land in anticipation of a bumper harvest each year. It therefore seems tragic to disregard a tool that has already been developed while the poor and the vulnerable communities suffer and depends on donor aid for survival. Notwithstanding genetic modification as the only strategy available to address agricultural problems, other strategies are failing, or not working fast enough in sync with climate change, population expansion and the devastating effect of pests and diseases, especially in Southern Africa. Using all the tools available to humankind in a comprehensive manner seems to be the best way to approach agricultural productivity problems and improve food security. Going forward, engaging farmers, industry, academic and public research sectors in collaborative discussions on the potential of GMOs will improve communication and enable effective policy discussions. In order to tap into the potential that biotechnology has and increase agricultural productivity and food security, there is a need for greater dedication by Southern Africa region toward technology development, harmonization of regulatory frameworks and dissociate politics from the science of GM technology. Furthermore, SADC should collaborate with other regional economic blocs such as Common Market for East and Southern Africa (COMESA), the Economic Community of West African States (ECOWAS) for harmonization of GMO regulation. As the clock for achieving the SDGs in 2030 is ticking away, Southern African governments need to define their own priorities for achieving and attaining sustainable development goals of eradicating poverty and hunger.
